# Hexa-μ_2_-chlorido-μ_4_-oxido-tetra­kis[(morpholine-κ*N*)copper(II)] methanol disolvate

**DOI:** 10.1107/S160053681401407X

**Published:** 2014-06-21

**Authors:** Kateryna Gubina, Vladimir Ovchynnikov, Vladimir Amirkhanov

**Affiliations:** a Department of Inorganic Chemistry, Kiev National Taras Shevchenko University, Vladimirskaya St. 64/13, Kiev 01601, Ukraine

**Keywords:** Tetra­nuclear copper(II) complex, morpholine, X-Ray, crystal structure

## Abstract

In the title solvate, [Cu_4_(μ_2_-Cl)_6_(μ_4_-O)(C_4_H_9_NO)_4_]·2CH_3_OH, each Cu^2+^ ion in the tetra­nuclear complex has a trigonal–bipyramidal coordination arising from three bridging chloride ions in equatorial positions and the central μ_4_-O^2−^ ion and morpholine N atom in axial positions. The morpholine rings adopt chair conformations, with the N—Cu bonds in equatorial orientations. In the crystal, the components are linked by N—H⋯O and O—H⋯O and O—H⋯Cl hydrogen bonds, which generate a three-dimensional network. One methanol mol­ecule is disordered over two sets of sites in a 0.642 (9):0.358 (9) ratio.

## Related literature   

For the chemistry and properties of polynuclear copper(II) complexes, see: Bertrand & Kelley (1966 ▶); Pavlenko *et al.* (1993 ▶); Linert *et al.* (1993 ▶); Bowmaker *et al.* (2011 ▶). For their role in the redox processes of biological systems, see: Erecinska & Wilson (1978 ▶). For details of the synthesis and structure of bis­(*N*,*N*′-morpholido)-[(*N*"-morpholido)-carboxamido]phosphate, see: Gubina *et al.* (1999 ▶). For the synthesis and structural investigation of copper–oxygen clusters and related materials, see: Weinberger *et al.* (1998 ▶); Roy & Manassero (2010 ▶); Bowmaker *et al.* (2011 ▶); Chivers *et al.* (2005 ▶); Li *et al.* (2011 ▶); Willett (1991 ▶). For standard copper–copper bond lengths, see: van Niekerk & Schoening (1953 ▶).
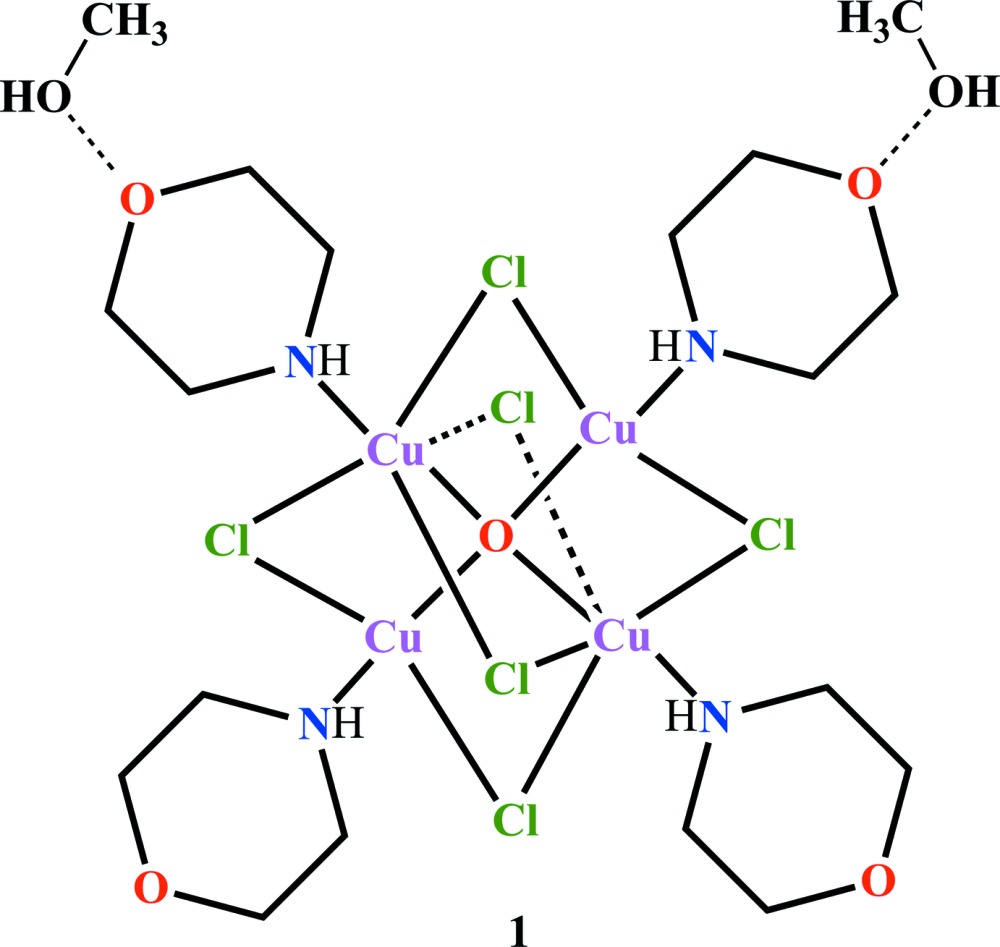


## Experimental   

### 

#### Crystal data  

[Cu_4_Cl_6_O(C_4_H_9_NO)_4_]·2CH_4_O
*M* *_r_* = 895.43
Monoclinic, 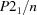

*a* = 11.149 (2) Å
*b* = 15.753 (3) Å
*c* = 18.905 (4) Å
β = 92.50 (3)°
*V* = 3317.1 (11) Å^3^
*Z* = 4
Mo *K*α radiation
μ = 3.05 mm^−1^
*T* = 100 K
0.25 × 0.25 × 0.20 mm

#### Data collection  

Kuma/Oxford Instruments KM4 diffractometer
Absorption correction: analytical (*CrysAlis RED*; UNILIC & Kuma Diffraction, 2000 ▶) *T*_min_ = 0.516, *T*_max_ = 0.580
18695 measured reflections
5761 independent reflections
4330 reflections with *I* > 2σ(*I*)
*R*_int_ = 0.069
5 standard reflections every 300 reflections intensity decay: 1.2%

#### Refinement  

*R*[*F* ^2^ > 2σ(*F* ^2^)] = 0.054
*wR*(*F* ^2^) = 0.092
*S* = 1.05
5761 reflections
381 parameters
6 restraints
H atoms treated by a mixture of independent and constrained refinement
Δρ_max_ = 0.45 e Å^−3^
Δρ_min_ = −0.55 e Å^−3^

### 

Data collection: *KM-4-CCD Software* (UNILIC & Kuma Diffraction, 2000 ▶); cell refinement: *KM-4-CCD Software*; data reduction: *KM-4-CCD Software*; program(s) used to solve structure: *SHELXS97* (Sheldrick, 2008 ▶); program(s) used to refine structure: *SHELXL97* (Sheldrick, 2008 ▶); molecular graphics: *ORTEP-3 for Windows* (Farrugia, 2012 ▶); software used to prepare material for publication: *WinGX* (Farrugia, 2012 ▶).

## Supplementary Material

Crystal structure: contains datablock(s) I. DOI: 10.1107/S160053681401407X/hb7241sup1.cif

Structure factors: contains datablock(s) I. DOI: 10.1107/S160053681401407X/hb7241Isup2.hkl

CCDC reference: 1008302

Additional supporting information:  crystallographic information; 3D view; checkCIF report

## Figures and Tables

**Table 1:** Selected bond lengths (Å)

Cu1—O1	1.906 (3)
Cu1—N1	1.981 (5)
Cu1—Cl3	2.4159 (16)
Cu1—Cl1	2.4224 (16)
Cu1—Cl2	2.4339 (17)
Cu2—O1	1.906 (4)
Cu2—N2	1.971 (5)
Cu2—Cl5	2.3917 (17)
Cu2—Cl3	2.4386 (16)
Cu2—Cl4	2.4478 (17)
Cu3—O1	1.910 (4)
Cu3—N3	1.983 (5)
Cu3—Cl2	2.4011 (16)
Cu3—Cl6	2.4124 (16)
Cu3—Cl4	2.4888 (15)
Cu4—O1	1.907 (3)
Cu4—N4	1.985 (5)
Cu4—Cl5	2.3788 (16)
Cu4—Cl6	2.3962 (17)
Cu4—Cl1	2.4312 (16)

**Table 2:** Hydrogen-bond geometry (Å, °)

*D*—H⋯*A*	*D*—H	H⋯*A*	*D*⋯*A*	*D*—H⋯*A*
N1—H1⋯O5^i^	0.84 (3)	2.22 (3)	3.060 (6)	177 (5)
N2—H2⋯O6^ii^	0.84 (3)	2.18 (3)	2.987 (8)	161 (5)
N3—H3⋯O8^iii^	0.84 (3)	2.05 (3)	2.871 (6)	167 (5)
N4—H4⋯O7^iv^	0.84 (3)	2.30 (3)	3.121 (12)	165 (5)
O7—H7*C*⋯O4	0.82	1.92	2.531 (12)	130
O8—H8⋯O2	0.82	1.91	2.724 (6)	173
O6—H6*C*⋯Cl4^v^	0.82	2.39	3.209 (7)	177

Symmetry codes: (i) 
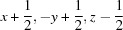
; (ii) 
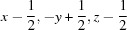
; (iii) 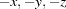; (iv) 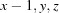; (v) 
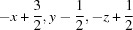
.

**Fig. 1.**
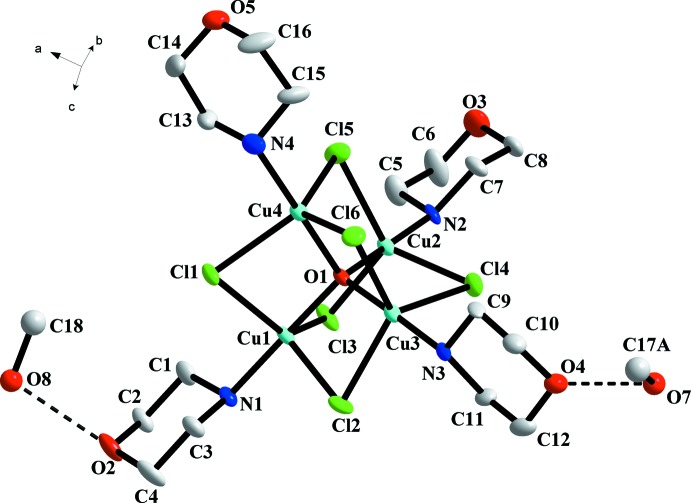
View of molecule [Cu4(µ4-O)(µ2-Cl)6(C4H9NO)4]·2CH3OH]. All H atoms have been omitted.

**Fig. 2.**
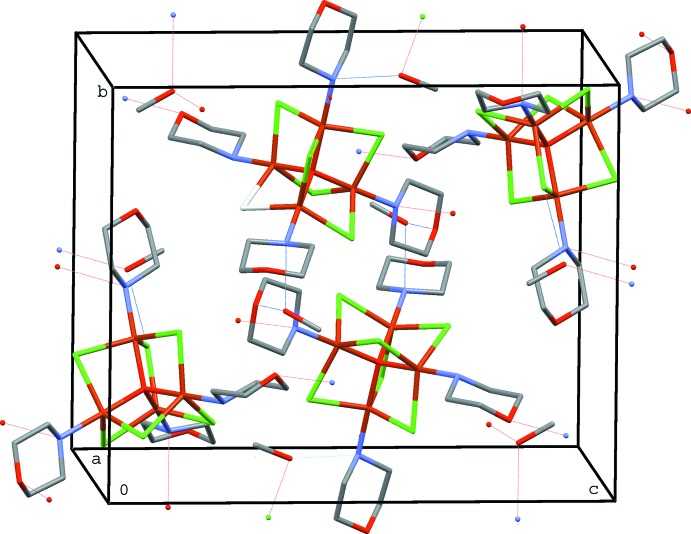
Packing view diagram of (1).

**Fig. 3.**
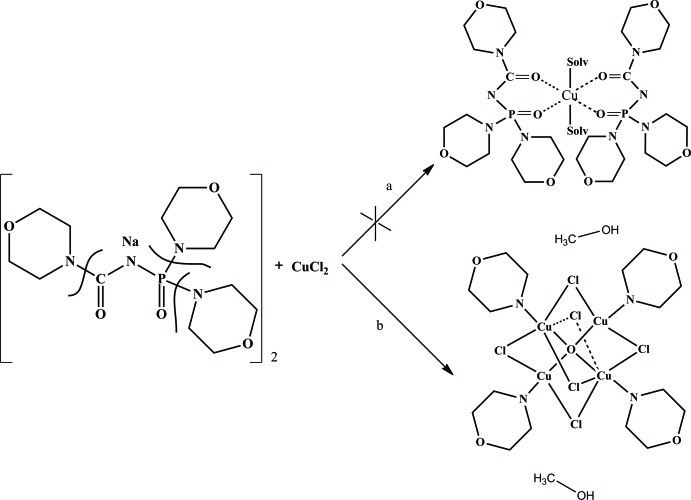
Scheme of the reaction.

## supplementary crystallographic information

### S1. Introduction

Polynuclear copper (II) complexes have been known for a long time and studied comprehensively (Bertrand *et al.*, 1966; Pavlenko *et al.*, 1993; Linert *et al.*, 1993; Bowmaker *et al.*, 2011). On the one hand they play significant role in the redox processes of biological systems (Erecinska *et al.*, 1978) and exhibit an inter­esting pattern of magnetic and electronic inter­actions in the copper-oxygen cluster (Willett *et al.*, 1991, Chivers *et al.*, 2005, Li *et al.*, 2011). On the other hand in the case of systems involving copper (II) salts and nitro­gen bases the presence of air and moisture leads to formation of occasionally crystallizing copper (II) substances. (Weinberger *et al.*, 1998, Roy *et al.*, 2010). They are inter­esting in their own right, providing some important model compounds, subjected to subsequent 'rational' synthesis (Bowmaker *et al.*, 2011). Herein we describe the structure of such kind complex of the general composition [Cu_4_OCl_6_(C_4_H_9_NO)_4_]·2CH_3_OH and compare with the similar acetone containing coordination compound, that was published by Weinberger *et al.*, 1998.

### S2. Experimental

All chemicals were commercial products of reagent grade and were used without further purification. Solvents were used as supplied or were distilled using standard methods.

Elemental analysis (C, H, N) was carried out on an Elementar Vario Micro Cube elemental analyzer. Cu ion was determined using of Perkin–Elmer AAS Analyst 400.

IR spectra were recorded using KBr pellets on a Perkin–Elmer Spectrum BX FTIR spectrophotometer in the range of 4000 to 400 cm^-1^.

Initial ligand (HL= OC_4_H_8_NC(O)NHP(O)(C_4_H_8_NO)_2_) (Scheme, Fig.3) for the synthesis of (I) was prepared according to the method of (Gubina *et al.*, 1999). The sodium salt NaL was obtained from a methanol solution by inter­action of HL with sodium methoxide in equimolar ratio. An expected complex with the composition [Cu(L)_2_], has to be obtained by an exchange reaction according Scheme (a). Solutions of NaL (2mmol) in methanol (10ml) with a solution of hydrated copper(II) chloride (1mmol) in methanol (15ml) were mixed. The resulted light green solution turned brown after a while. The composition of the precipitated substance was significantly different from the calculated for the complex [Cu(L)_2_]. Theoretically calculated for [Cu(L)_2_], C_26_H_48_N_8_O_10_P_2_Cu: C, 41.19%; H, 6.38%; N,14.78%; Cu, 8.38%.Found: C, 21.95%; H, 4.07%; N, 5.17 %; Cu, 25.5%. Theoretically calculated for [Cu_4_OCl_6_(C_4_H_9_NO)_4_]· 2CH_3_OH, C_18_H_44_Cl_6_Cu_4_N_4_O_7_: C, 21.58%; H, 4.43%; N,5.59 %; Cu, 25.37%.

Most likely the phospho­rylated carbamide ligand was destruct under catalytic influence of a copper ion and moisture of air. The released morpholine molecules formed the new copper (II) coordination compound of the general formula [Cu_4_OCl_6_(C_4_H_9_NO)_4_]·2CH_3_OH (Scheme b). The product was filtered out, washed with cold methanol and dried in desiccator under CaCl_2_ (yield 67%). The compound was recrystallised from methanol yielding brown blocks of an methanol solvate [Cu_4_OCl_6_(C_4_H_9_NO)_4_]·2CH_3_OH. The crystals slowly lost the solvent at room temperature. IR spectra of obtained compound (I) show the absence of C=O and P=O bands. IR (KBr): δ_s_(CH_2_) 1400 vs, δ_as_(CH_2_) 1245s, ν(CN) 1040 vs, δ_s_(CNC) 600 vs, ν(Cu_4_O) 580s, ν(CuN) 443m, cm^-1^.

### S2.1. Synthesis and crystallization

Slow crystallization from methanol.

### S2.2. Refinement

Crystal data, data collection and structure refinement details are summarized in Table 1. All H atoms of methyl methanol molecules and methyl­ene groups of morpholine rings were calculated geometrically and subsequently treated as riding model, with C—H = 0.98 (methyl) C—H = 0.98 (methyl­ene), U_iso_(H) = 1.5U_eq_(C) U_iso_(H) = 1.2U_eq_(C) respectively. H atoms of OH group of methanol molecules were detected in a difference Fourier and further refined with O—H = 0.82Å and subsequently treated as riding model with U_iso_(H) = 1.2U_eq_(O). H atoms of the amine group were located in a difference Fourier map and further refined with similarity restraints for d(N—H) and Uiso(H) = 1.2Ueq(N). One methanol molecule is disordered, with occupancies of 0.642 (9) and 0.358 (9).

### S3. Results and discussion

Cu_4_(*µ_4_*-O)(*µ_2_*-Cl)_6_(C_4_H_9_NO)_4_]·2CH_3_OH (1) crystallizes in the centrosymmetric space group P2(1)/n with unit cell containing one independent molecule of tetra­nuclear Cu complex and two methanol solvent molecules (Fig.1). All copper ions have trigonal bipyramidal coordination figures with three Cl atoms in equatorial positions and *µ_4_*-O and N in axial positions. This complex displays the difference with many other Cu_4_(*µ_4_*-O)(*µ_2_*-halogen)_6_L_4_ complexes reported in CCDC and also described in (Weinberger *et al.*, 1998). The presently investigated hexa-chloro morpholine species can be expected to have a similar molecular structure like in published earlier Cu_4_(*µ_4_*-O)(*µ_2_*-Cl)_6_(C_4_H_9_NO)_4_]·1/3(CH_3_)_2_CO (2) (Weinberger *et al.*, 1998); however the unit cell of (1) containing one independent molecule of complex against in (2). Structure (1) has two molecules of methanol, one of them is disordered over two positions with degree of filling 0.64. Unlike the title compound (2) the structure (1) has the OH protons of methanol connected by hydrogen bonds with oxygen atoms of morpholine rings (Fig.2). Important bond lengths for (1) are shown in the Table 2. The distances Cu—O are 1.907Å in average and metal-metal inter­atomic contacts are approximately 3.110 (1)Å, which is longer than the value for standard copper-copper bonds (2.64Å) (van Niekerk *et al.*, 1953). The angles values Cu—O—Cu 109.82 (18)Å suggest sp^3^-hybridization of the oxygen orbital.

The values of inter­atomic distances Cu—N, Cu—O and Cu—Cl agree well with reported ones for known complexes (Weinberger *et al.*, 1998).

A packing diagram of the compound (see Fig.2) reveals that due to various types of hydrogen bonds the 3D polymer is formed. All four hydrogen atoms of the NH groups of the morpholine rings form straight N—H···O (Cl) hydrogen bonds (Table 3). In addition each of desorded OH group of methanol also involved in the formation of hydrogen bonds system. The morpholine residues exhibit orientations relative to the [Cu_4_(*µ_4_*-O)(*µ_2_*-Cl)_6_] cores by which the Cu-bonded morpholine NH groups point with their N—H vectors halfway between two neighboring Cl ions. The methanol molecules do not inter­act directly with one of the copper coordination centers of the halide bridges.

We have noticed that the losses of methanol cause a decrease in crystalline of the substanstance as well as in (2) (Weinberger *et al.*, 1998, Roy *et al.*, 2010) .

Solvate formation is relatively common in the family of the tetra­nuclear Cu—O-*hal* complexes and has been reported for more than 50 of the known crystal structures included in CCDC. At the end it must be noted that the [Cu_4_(*µ_4_*-O)(*µ_2_*-Cl)_6_] core is quiet stable and is formed both as in the target synthesis, so as a side product of the various type reactions.

### Crystal data

**Table:**

[Cu_4_Cl_6_O(C_4_H_9_NO)_4_]·2CH_4_O	*F*(000) = 1816
*M**_r_* = 895.43	*D*_x_ = 1.793 Mg m^−^^3^
Monoclinic, *P*2_1_/*n*	Mo *K*α radiation, λ = 0.71073 Å
Hall symbol: -P 2yn	Cell parameters from 23105 reflections
*a* = 11.149 (2) Å	θ = 3.4–25.0°
*b* = 15.753 (3) Å	µ = 3.05 mm^−^^1^
*c* = 18.905 (4) Å	*T* = 100 K
β = 92.50 (3)°	Block, brown
*V* = 3317.1 (11) Å^3^	0.25 × 0.25 × 0.20 mm
*Z* = 4	

### Data collection

**Table:**

Kuma/Oxford Instruments KM4 diffractometer	4330 reflections with *I* > 2σ(*I*)
Radiation source: fine-focus sealed tube	*R*_int_ = 0.069
Graphite monochromator	θ_max_ = 25.0°, θ_min_ = 3.4°
ω scans	*h* = −13→8
Absorption correction: analytical (*CrysAlis RED*; UNILIC & Kuma Diffraction, 2000)	*k* = −18→18
*T*_min_ = 0.516, *T*_max_ = 0.580	*l* = −22→22
18695 measured reflections	5 standard reflections every 300 reflections
5761 independent reflections	intensity decay: 1.2%

### Refinement

**Table:**

Refinement on *F*^2^	Primary atom site location: structure-invariant direct methods
Least-squares matrix: full	Secondary atom site location: difference Fourier map
*R*[*F*^2^ > 2σ(*F*^2^)] = 0.054	Hydrogen site location: inferred from neighbouring sites
*wR*(*F*^2^) = 0.092	H atoms treated by a mixture of independent and constrained refinement
*S* = 1.05	*w* = 1/[σ^2^(*F*_o_^2^) + (0.0306*P*)^2^] where *P* = (*F*_o_^2^ + 2*F*_c_^2^)/3
5761 reflections	(Δ/σ)_max_ < 0.001
381 parameters	Δρ_max_ = 0.45 e Å^−^^3^
6 restraints	Δρ_min_ = −0.55 e Å^−^^3^

### Special details

**Table:**

Geometry. All s.u.'s (except the s.u. in the dihedral angle between two l.s. planes) are estimated using the full covariance matrix. The cell s.u.'s are taken into account individually in the estimation of s.u.'s in distances, angles and torsion angles; correlations between s.u.'s in cell parameters are only used when they are defined by crystal symmetry. An approximate (isotropic) treatment of cell s.u.'s is used for estimating s.u.'s involving l.s. planes.
Refinement. Refinement of *F*^2^ against ALL reflections. The weighted *R*-factor *wR* and goodness of fit *S* are based on *F*^2^, conventional *R*-factors *R* are based on *F*, with *F* set to zero for negative *F*^2^. The threshold expression of *F*^2^ > 2σ(*F*^2^) is used only for calculating *R*-factors(gt) *etc*. and is not relevant to the choice of reflections for refinement. *R*-factors based on *F*^2^ are statistically about twice as large as those based on *F*, and *R*- factors based on ALL data will be even larger.

### Fractional atomic coordinates and isotropic or equivalent isotropic displacement parameters (Å^2^)

**Table:**

	*x*	*y*	*z*	*U*_iso_*/*U*_eq_	Occ. (<1)
Cu1	0.11029 (6)	0.20266 (4)	−0.00488 (3)	0.01943 (18)	
Cl1	−0.06439 (12)	0.16905 (9)	0.06165 (7)	0.0231 (3)	
O1	0.1733 (3)	0.2528 (2)	0.08065 (17)	0.0182 (8)	
N1	0.0461 (4)	0.1520 (3)	−0.0946 (2)	0.0202 (11)	
H1	0.105 (4)	0.153 (4)	−0.120 (3)	0.024*	
C1	−0.0588 (5)	0.1985 (4)	−0.1265 (3)	0.0243 (14)	
H1B	−0.0358	0.2569	−0.1350	0.029*	
H1A	−0.1224	0.1991	−0.0931	0.029*	
Cu2	0.21716 (6)	0.36779 (4)	0.06401 (3)	0.01899 (18)	
Cl2	0.27880 (14)	0.10492 (9)	0.00872 (7)	0.0281 (4)	
O2	−0.1338 (4)	0.0715 (2)	−0.18659 (19)	0.0304 (10)	
N2	0.2632 (4)	0.4869 (3)	0.0481 (2)	0.0204 (11)	
H2	0.317 (4)	0.485 (4)	0.018 (2)	0.025*	
C2	−0.1063 (5)	0.1598 (4)	−0.1953 (3)	0.0254 (15)	
H2B	−0.1782	0.1899	−0.2117	0.030*	
H2A	−0.0469	0.1660	−0.2309	0.030*	
Cu3	0.30689 (6)	0.18666 (4)	0.11536 (3)	0.02035 (18)	
Cl3	0.15197 (14)	0.33820 (9)	−0.05832 (7)	0.0301 (4)	
N3	0.4437 (4)	0.1155 (3)	0.1505 (2)	0.0192 (11)	
H3	0.416 (5)	0.067 (2)	0.142 (3)	0.023*	
O3	0.2561 (4)	0.6693 (3)	0.0666 (2)	0.0381 (11)	
C3	0.0162 (5)	0.0602 (4)	−0.0896 (3)	0.0265 (15)	
H3B	−0.0442	0.0523	−0.0548	0.032*	
H3A	0.0874	0.0288	−0.0739	0.032*	
Cu4	0.05148 (6)	0.25276 (4)	0.14837 (3)	0.01961 (18)	
Cl4	0.41882 (12)	0.32233 (9)	0.10401 (8)	0.0244 (3)	
N4	−0.0769 (5)	0.2534 (3)	0.2179 (3)	0.0274 (12)	
H4	−0.096 (5)	0.203 (2)	0.228 (3)	0.033*	
O4	0.6864 (3)	0.0843 (2)	0.2060 (2)	0.0274 (10)	
C4	−0.0305 (6)	0.0258 (4)	−0.1606 (3)	0.0339 (17)	
H4B	0.0320	0.0299	−0.1946	0.041*	
H4A	−0.0510	−0.0337	−0.1556	0.041*	
Cl5	0.05980 (14)	0.40336 (9)	0.14077 (8)	0.0308 (4)	
O5	−0.2421 (4)	0.3385 (3)	0.3081 (2)	0.0302 (10)	
C5	0.1673 (6)	0.5418 (4)	0.0141 (3)	0.0340 (17)	
H5B	0.0988	0.5435	0.0439	0.041*	
H5A	0.1411	0.5175	−0.0311	0.041*	
Cl6	0.18960 (13)	0.16776 (9)	0.21846 (7)	0.0247 (3)	
C6	0.2130 (6)	0.6318 (4)	0.0027 (4)	0.046 (2)	
H6B	0.2770	0.6304	−0.0304	0.055*	
H6A	0.1484	0.6662	−0.0179	0.055*	
C7	0.3103 (5)	0.5307 (4)	0.1141 (3)	0.0256 (14)	
H7B	0.3770	0.4986	0.1350	0.031*	
H7A	0.2478	0.5332	0.1481	0.031*	
C8	0.3514 (5)	0.6196 (4)	0.0976 (3)	0.0265 (15)	
H8B	0.3817	0.6466	0.1408	0.032*	
H8A	0.4165	0.6168	0.0652	0.032*	
O8	−0.3635 (4)	0.0576 (3)	−0.1444 (2)	0.0350 (11)	
H8	−0.2966	0.0612	−0.1606	0.042*	
C9	0.4804 (5)	0.1266 (4)	0.2268 (3)	0.0256 (15)	
H9B	0.4129	0.1134	0.2556	0.031*	
H9A	0.5024	0.1854	0.2355	0.031*	
C10	0.5853 (5)	0.0699 (4)	0.2485 (3)	0.0272 (15)	
H10B	0.6085	0.0804	0.2978	0.033*	
H10A	0.5607	0.0110	0.2441	0.033*	
C11	0.5512 (5)	0.1222 (4)	0.1067 (3)	0.0237 (14)	
H11B	0.5788	0.1806	0.1068	0.028*	
H11A	0.5290	0.1068	0.0582	0.028*	
C12	0.6530 (5)	0.0652 (4)	0.1339 (3)	0.0277 (15)	
H12B	0.6281	0.0064	0.1301	0.033*	
H12A	0.7219	0.0730	0.1049	0.033*	
C13	−0.1891 (5)	0.2937 (4)	0.1901 (3)	0.0332 (16)	
H13B	−0.2201	0.2624	0.1491	0.040*	
H13A	−0.1722	0.3512	0.1751	0.040*	
C14	−0.2840 (5)	0.2957 (5)	0.2465 (4)	0.0435 (19)	
H14B	−0.3557	0.3238	0.2272	0.052*	
H14A	−0.3055	0.2380	0.2586	0.052*	
C15	−0.0377 (6)	0.2934 (4)	0.2863 (3)	0.0327 (16)	
H15B	−0.0116	0.3511	0.2778	0.039*	
H15A	0.0299	0.2621	0.3072	0.039*	
C16	−0.1405 (6)	0.2944 (4)	0.3381 (3)	0.0400 (18)	
H16B	−0.1631	0.2365	0.3491	0.048*	
H16A	−0.1135	0.3218	0.3818	0.048*	
C18	−0.3645 (6)	0.1021 (4)	−0.0787 (3)	0.0436 (19)	
H18C	−0.3501	0.1613	−0.0866	0.065*	
H18B	−0.3028	0.0798	−0.0469	0.052*	
H18A	−0.4413	0.0950	−0.0583	0.052*	
C17	0.871 (3)	0.0241 (16)	0.3535 (18)	0.036 (4)	0.642 (9)
H17A	0.7852	0.0210	0.3501	0.054*	0.642 (9)
H17B	0.9039	−0.0070	0.3152	0.054*	0.642 (9)
H17C	0.8959	0.0824	0.3511	0.054*	0.642 (9)
O6	0.9179 (6)	−0.0147 (4)	0.4251 (3)	0.036 (2)	0.642 (9)
H6C	0.9622	−0.0549	0.4175	0.053*	0.642 (9)
C17A	0.852 (6)	0.003 (3)	0.349 (4)	0.036 (4)	0.358 (9)
H17D	0.7676	−0.0005	0.3550	0.054*	0.358 (9)
H17E	0.8920	0.0227	0.3919	0.054*	0.358 (9)
H17F	0.8829	−0.0514	0.3365	0.054*	0.358 (9)
O7	0.8786 (10)	0.0741 (8)	0.2819 (7)	0.038 (4)	0.358 (9)
H7C	0.8181	0.1026	0.2734	0.045*	0.358 (9)

### Atomic displacement parameters (Å^2^)

**Table:**

	*U*^11^	*U*^22^	*U*^33^	*U*^12^	*U*^13^	*U*^23^
Cu1	0.0277 (4)	0.0189 (4)	0.0118 (4)	−0.0113 (3)	0.0023 (3)	−0.0033 (3)
Cl1	0.0271 (8)	0.0264 (8)	0.0159 (7)	−0.0160 (7)	0.0033 (6)	−0.0031 (6)
O1	0.023 (2)	0.020 (2)	0.012 (2)	−0.0105 (18)	0.0032 (16)	−0.0037 (16)
N1	0.026 (3)	0.023 (3)	0.013 (3)	−0.009 (2)	0.001 (2)	−0.001 (2)
C1	0.033 (4)	0.021 (3)	0.019 (3)	−0.013 (3)	−0.001 (3)	0.003 (3)
Cu2	0.0236 (4)	0.0188 (4)	0.0150 (4)	−0.0094 (3)	0.0043 (3)	−0.0024 (3)
Cl2	0.0393 (9)	0.0273 (9)	0.0175 (8)	−0.0001 (7)	−0.0012 (7)	−0.0093 (6)
O2	0.051 (3)	0.019 (2)	0.020 (2)	−0.018 (2)	−0.008 (2)	0.0024 (18)
N2	0.024 (3)	0.022 (3)	0.016 (3)	−0.014 (2)	0.000 (2)	−0.005 (2)
C2	0.037 (4)	0.022 (3)	0.016 (3)	−0.013 (3)	−0.004 (3)	0.001 (3)
Cu3	0.0214 (4)	0.0258 (4)	0.0142 (4)	−0.0092 (3)	0.0039 (3)	−0.0043 (3)
Cl3	0.0488 (10)	0.0272 (9)	0.0140 (8)	−0.0230 (8)	−0.0028 (7)	0.0018 (6)
N3	0.018 (3)	0.023 (3)	0.017 (3)	−0.015 (2)	0.007 (2)	−0.005 (2)
O3	0.042 (3)	0.018 (2)	0.054 (3)	−0.002 (2)	−0.001 (2)	0.000 (2)
C3	0.036 (4)	0.022 (4)	0.021 (3)	−0.009 (3)	0.000 (3)	−0.005 (3)
Cu4	0.0234 (4)	0.0209 (4)	0.0150 (4)	−0.0086 (3)	0.0061 (3)	−0.0023 (3)
Cl4	0.0237 (8)	0.0180 (8)	0.0314 (9)	−0.0071 (6)	−0.0004 (7)	0.0023 (6)
N4	0.035 (3)	0.020 (3)	0.028 (3)	−0.010 (3)	0.011 (2)	−0.002 (2)
O4	0.029 (2)	0.027 (2)	0.026 (2)	0.002 (2)	0.004 (2)	−0.0033 (19)
C4	0.063 (5)	0.017 (3)	0.021 (4)	−0.012 (3)	−0.008 (3)	−0.002 (3)
Cl5	0.0355 (9)	0.0200 (8)	0.0385 (9)	−0.0065 (7)	0.0196 (8)	0.0009 (7)
O5	0.037 (3)	0.029 (2)	0.026 (2)	0.002 (2)	0.010 (2)	−0.002 (2)
C5	0.039 (4)	0.025 (4)	0.037 (4)	−0.007 (3)	−0.012 (3)	0.008 (3)
Cl6	0.0299 (8)	0.0292 (9)	0.0156 (7)	−0.0014 (7)	0.0085 (6)	0.0040 (6)
C6	0.053 (5)	0.028 (4)	0.054 (5)	−0.016 (4)	−0.023 (4)	0.014 (4)
C7	0.038 (4)	0.022 (3)	0.017 (3)	−0.010 (3)	0.002 (3)	−0.006 (3)
C8	0.030 (4)	0.020 (4)	0.029 (4)	−0.010 (3)	0.001 (3)	−0.009 (3)
O8	0.046 (3)	0.034 (3)	0.025 (2)	−0.022 (2)	0.002 (2)	−0.005 (2)
C9	0.023 (3)	0.045 (4)	0.010 (3)	−0.010 (3)	0.007 (3)	−0.014 (3)
C10	0.033 (4)	0.031 (4)	0.018 (3)	−0.004 (3)	0.005 (3)	−0.003 (3)
C11	0.024 (3)	0.027 (4)	0.021 (3)	−0.002 (3)	0.010 (3)	−0.003 (3)
C12	0.036 (4)	0.020 (3)	0.028 (4)	0.002 (3)	0.012 (3)	−0.001 (3)
C13	0.025 (4)	0.052 (4)	0.023 (4)	−0.006 (3)	0.003 (3)	−0.015 (3)
C14	0.025 (4)	0.051 (5)	0.056 (5)	−0.015 (3)	0.016 (3)	−0.024 (4)
C15	0.043 (4)	0.036 (4)	0.020 (3)	0.020 (3)	0.008 (3)	0.002 (3)
C16	0.057 (5)	0.041 (4)	0.023 (4)	0.023 (4)	0.020 (3)	0.013 (3)
C18	0.059 (5)	0.044 (5)	0.028 (4)	−0.019 (4)	0.006 (4)	−0.006 (3)
C17	0.024 (11)	0.015 (13)	0.069 (7)	−0.009 (8)	0.001 (7)	−0.009 (11)
O6	0.034 (4)	0.038 (5)	0.035 (5)	0.012 (3)	0.013 (3)	0.001 (3)
C17A	0.024 (11)	0.015 (13)	0.069 (7)	−0.009 (8)	0.001 (7)	−0.009 (11)
O7	0.035 (8)	0.032 (8)	0.044 (9)	−0.004 (6)	−0.004 (6)	0.013 (6)

### Geometric parameters (Å, º)

**Table:**

Cu1—Cu3	3.0984 (13)	C4—H4B	0.9700
Cu1—Cu4	3.1002 (11)	C4—H4A	0.9700
Cu2—Cu4	3.0816 (11)	O5—C14	1.408 (7)
Cu2—Cu3	3.1623 (11)	O5—C16	1.425 (7)
Cu1—O1	1.906 (3)	C5—C6	1.525 (8)
Cu1—N1	1.981 (5)	C5—H5B	0.9700
Cu1—Cl3	2.4159 (16)	C5—H5A	0.9700
Cu1—Cl1	2.4224 (16)	C6—H6B	0.9700
Cu1—Cl2	2.4339 (17)	C6—H6A	0.9700
Cu2—O1	1.906 (4)	C7—C8	1.510 (8)
Cu2—N2	1.971 (5)	C7—H7B	0.9700
Cu2—Cl5	2.3917 (17)	C7—H7A	0.9700
Cu2—Cl3	2.4386 (16)	C8—H8B	0.9700
Cu2—Cl4	2.4478 (17)	C8—H8A	0.9700
Cu3—O1	1.910 (4)	O8—C18	1.427 (7)
Cu3—N3	1.983 (5)	O8—H8	0.8200
Cu3—Cl2	2.4011 (16)	C9—C10	1.514 (8)
Cu3—Cl6	2.4124 (16)	C9—H9B	0.9700
Cu3—Cl4	2.4888 (15)	C9—H9A	0.9700
Cu4—O1	1.907 (3)	C10—H10B	0.9700
Cu4—N4	1.985 (5)	C10—H10A	0.9700
Cu4—Cl5	2.3788 (16)	C11—C12	1.519 (7)
Cu4—Cl6	2.3962 (17)	C11—H11B	0.9700
Cu4—Cl1	2.4312 (16)	C11—H11A	0.9700
N1—C1	1.486 (7)	C12—H12B	0.9700
N1—C3	1.488 (7)	C12—H12A	0.9700
N1—H1	0.84 (3)	C13—C14	1.535 (8)
C1—C2	1.511 (7)	C13—H13B	0.9700
C1—H1B	0.9700	C13—H13A	0.9700
C1—H1A	0.9700	C14—H14B	0.9700
O2—C4	1.427 (7)	C14—H14A	0.9700
O2—C2	1.437 (6)	C15—C16	1.540 (8)
N2—C5	1.499 (7)	C15—H15B	0.9700
N2—C7	1.500 (6)	C15—H15A	0.9700
N2—H2	0.84 (3)	C16—H16B	0.9700
C2—H2B	0.9700	C16—H16A	0.9700
C2—H2A	0.9700	C18—H18C	0.9600
N3—C11	1.490 (6)	C18—H18B	0.9600
N3—C9	1.494 (7)	C18—H18A	0.9600
N3—H3	0.84 (3)	C17—O6	1.55 (4)
O3—C6	1.410 (7)	C17—H17A	0.9600
O3—C8	1.425 (7)	C17—H17B	0.9600
C3—C4	1.520 (7)	C17—H17C	0.9600
C3—H3B	0.9700	O6—H6C	0.8200
C3—H3A	0.9700	C17A—O7	1.72 (6)
N4—C13	1.479 (8)	C17A—H17D	0.9600
N4—C15	1.485 (7)	C17A—H17E	0.9600
N4—H4	0.84 (3)	C17A—H17F	0.9600
O4—C12	1.429 (6)	O7—H7C	0.8200
O4—C10	1.430 (6)		
			
O1—Cu1—N1	179.09 (17)	O1—Cu4—Cu2	36.08 (11)
O1—Cu1—Cl3	85.30 (11)	N4—Cu4—Cu2	143.64 (15)
N1—Cu1—Cl3	93.84 (14)	Cl5—Cu4—Cu2	49.94 (4)
O1—Cu1—Cl1	85.71 (11)	Cl6—Cu4—Cu2	103.27 (4)
N1—Cu1—Cl1	95.05 (14)	Cl1—Cu4—Cu2	106.43 (4)
Cl3—Cu1—Cl1	125.72 (6)	O1—Cu4—Cu1	35.59 (10)
O1—Cu1—Cl2	85.30 (12)	N4—Cu4—Cu1	143.93 (15)
N1—Cu1—Cl2	94.80 (15)	Cl5—Cu4—Cu1	100.78 (4)
Cl3—Cu1—Cl2	116.21 (6)	Cl6—Cu4—Cu1	102.56 (5)
Cl1—Cu1—Cl2	116.18 (6)	Cl1—Cu4—Cu1	50.18 (4)
O1—Cu1—Cu3	35.75 (11)	Cu2—Cu4—Cu1	60.67 (3)
N1—Cu1—Cu3	144.42 (15)	Cu2—Cl4—Cu3	79.67 (5)
Cl3—Cu1—Cu3	103.64 (4)	C13—N4—C15	109.9 (5)
Cl1—Cu1—Cu3	99.23 (4)	C13—N4—Cu4	112.9 (4)
Cl2—Cu1—Cu3	49.68 (4)	C15—N4—Cu4	112.7 (4)
O1—Cu1—Cu4	35.61 (10)	C13—N4—H4	105 (4)
N1—Cu1—Cu4	145.08 (14)	C15—N4—H4	106 (4)
Cl3—Cu1—Cu4	102.60 (4)	Cu4—N4—H4	110 (4)
Cl1—Cu1—Cu4	50.43 (4)	C12—O4—C10	108.9 (4)
Cl2—Cu1—Cu4	104.84 (5)	O2—C4—C3	111.4 (5)
Cu3—Cu1—Cu4	60.45 (3)	O2—C4—H4B	109.4
Cu1—Cl1—Cu4	79.40 (5)	C3—C4—H4B	109.4
Cu1—O1—Cu2	109.96 (17)	O2—C4—H4A	109.4
Cu1—O1—Cu4	108.80 (16)	C3—C4—H4A	109.4
Cu2—O1—Cu4	107.83 (18)	H4B—C4—H4A	108.0
Cu1—O1—Cu3	108.59 (18)	Cu4—Cl5—Cu2	80.48 (5)
Cu2—O1—Cu3	111.93 (17)	C14—O5—C16	109.0 (5)
Cu4—O1—Cu3	109.68 (17)	N2—C5—C6	111.1 (5)
C1—N1—C3	109.3 (4)	N2—C5—H5B	109.4
C1—N1—Cu1	113.4 (3)	C6—C5—H5B	109.4
C3—N1—Cu1	114.2 (3)	N2—C5—H5A	109.4
C1—N1—H1	112 (4)	C6—C5—H5A	109.4
C3—N1—H1	104 (4)	H5B—C5—H5A	108.0
Cu1—N1—H1	103 (4)	Cu4—Cl6—Cu3	80.92 (5)
N1—C1—C2	113.0 (5)	O3—C6—C5	111.9 (5)
N1—C1—H1B	109.0	O3—C6—H6B	109.2
C2—C1—H1B	109.0	C5—C6—H6B	109.2
N1—C1—H1A	109.0	O3—C6—H6A	109.2
C2—C1—H1A	109.0	C5—C6—H6A	109.2
H1B—C1—H1A	107.8	H6B—C6—H6A	107.9
O1—Cu2—N2	179.25 (17)	N2—C7—C8	110.7 (5)
O1—Cu2—Cl5	85.55 (11)	N2—C7—H7B	109.5
N2—Cu2—Cl5	94.16 (15)	C8—C7—H7B	109.5
O1—Cu2—Cl3	84.66 (11)	N2—C7—H7A	109.5
N2—Cu2—Cl3	96.09 (14)	C8—C7—H7A	109.5
Cl5—Cu2—Cl3	115.09 (6)	H7B—C7—H7A	108.1
O1—Cu2—Cl4	84.81 (11)	O3—C8—C7	111.6 (5)
N2—Cu2—Cl4	94.80 (14)	O3—C8—H8B	109.3
Cl5—Cu2—Cl4	124.70 (6)	C7—C8—H8B	109.3
Cl3—Cu2—Cl4	117.96 (6)	O3—C8—H8A	109.3
O1—Cu2—Cu4	36.09 (10)	C7—C8—H8A	109.3
N2—Cu2—Cu4	143.56 (14)	H8B—C8—H8A	108.0
Cl5—Cu2—Cu4	49.58 (4)	C18—O8—H8	109.5
Cl3—Cu2—Cu4	102.58 (4)	N3—C9—C10	111.6 (5)
Cl4—Cu2—Cu4	103.37 (4)	N3—C9—H9B	109.3
O1—Cu2—Cu3	34.07 (10)	C10—C9—H9B	109.3
N2—Cu2—Cu3	145.52 (14)	N3—C9—H9A	109.3
Cl5—Cu2—Cu3	104.81 (4)	C10—C9—H9A	109.3
Cl3—Cu2—Cu3	101.29 (5)	H9B—C9—H9A	108.0
Cl4—Cu2—Cu3	50.74 (4)	O4—C10—C9	111.9 (5)
Cu4—Cu2—Cu3	59.95 (2)	O4—C10—H10B	109.2
Cu3—Cl2—Cu1	79.70 (5)	C9—C10—H10B	109.2
C4—O2—C2	110.8 (4)	O4—C10—H10A	109.2
C5—N2—C7	107.9 (5)	C9—C10—H10A	109.2
C5—N2—Cu2	115.4 (3)	H10B—C10—H10A	107.9
C7—N2—Cu2	113.3 (3)	N3—C11—C12	112.2 (5)
C5—N2—H2	104 (4)	N3—C11—H11B	109.2
C7—N2—H2	110 (4)	C12—C11—H11B	109.2
Cu2—N2—H2	106 (4)	N3—C11—H11A	109.2
O2—C2—C1	111.1 (4)	C12—C11—H11A	109.2
O2—C2—H2B	109.4	H11B—C11—H11A	107.9
C1—C2—H2B	109.4	O4—C12—C11	111.0 (5)
O2—C2—H2A	109.4	O4—C12—H12B	109.4
C1—C2—H2A	109.4	C11—C12—H12B	109.4
H2B—C2—H2A	108.0	O4—C12—H12A	109.4
O1—Cu3—N3	178.62 (17)	C11—C12—H12A	109.4
O1—Cu3—Cl2	86.15 (11)	H12B—C12—H12A	108.0
N3—Cu3—Cl2	92.75 (14)	N4—C13—C14	111.1 (5)
O1—Cu3—Cl6	84.43 (11)	N4—C13—H13B	109.4
N3—Cu3—Cl6	95.50 (14)	C14—C13—H13B	109.4
Cl2—Cu3—Cl6	123.83 (6)	N4—C13—H13A	109.4
O1—Cu3—Cl4	83.60 (11)	C14—C13—H13A	109.4
N3—Cu3—Cl4	97.65 (13)	H13B—C13—H13A	108.0
Cl2—Cu3—Cl4	115.83 (6)	O5—C14—C13	111.7 (5)
Cl6—Cu3—Cl4	117.85 (6)	O5—C14—H14B	109.3
O1—Cu3—Cu1	35.66 (10)	C13—C14—H14B	109.3
N3—Cu3—Cu1	143.16 (13)	O5—C14—H14A	109.3
Cl2—Cu3—Cu1	50.61 (4)	C13—C14—H14A	109.3
Cl6—Cu3—Cu1	102.22 (5)	H14B—C14—H14A	107.9
Cl4—Cu3—Cu1	102.02 (4)	N4—C15—C16	111.0 (5)
O1—Cu3—Cu2	34.00 (10)	N4—C15—H15B	109.4
N3—Cu3—Cu2	147.24 (13)	C16—C15—H15B	109.4
Cl2—Cu3—Cu2	101.43 (5)	N4—C15—H15A	109.4
Cl6—Cu3—Cu2	100.61 (4)	C16—C15—H15A	109.4
Cl4—Cu3—Cu2	49.60 (4)	H15B—C15—H15A	108.0
Cu1—Cu3—Cu2	59.82 (2)	O5—C16—C15	110.6 (5)
Cu1—Cl3—Cu2	80.05 (5)	O5—C16—H16B	109.5
C11—N3—C9	109.6 (4)	C15—C16—H16B	109.5
C11—N3—Cu3	113.6 (3)	O5—C16—H16A	109.5
C9—N3—Cu3	115.6 (4)	C15—C16—H16A	109.5
C11—N3—H3	105 (4)	H16B—C16—H16A	108.1
C9—N3—H3	112 (4)	O8—C18—H18C	109.5
Cu3—N3—H3	100 (4)	O8—C18—H18B	109.5
C6—O3—C8	109.9 (5)	H18C—C18—H18B	109.5
N1—C3—C4	111.0 (5)	O8—C18—H18A	109.5
N1—C3—H3B	109.4	H18C—C18—H18A	109.5
C4—C3—H3B	109.4	H18B—C18—H18A	109.5
N1—C3—H3A	109.4	O6—C17—H17A	109.5
C4—C3—H3A	109.4	O6—C17—H17B	109.5
H3B—C3—H3A	108.0	H17A—C17—H17B	109.5
O1—Cu4—N4	179.2 (2)	O6—C17—H17C	109.5
O1—Cu4—Cl5	85.90 (12)	H17A—C17—H17C	109.5
N4—Cu4—Cl5	93.77 (15)	H17B—C17—H17C	109.5
O1—Cu4—Cl6	84.94 (11)	O7—C17A—H17D	109.5
N4—Cu4—Cl6	95.81 (16)	O7—C17A—H17E	109.5
Cl5—Cu4—Cl6	124.39 (6)	H17D—C17A—H17E	109.5
O1—Cu4—Cl1	85.44 (11)	O7—C17A—H17F	109.5
N4—Cu4—Cl1	94.15 (15)	H17D—C17A—H17F	109.5
Cl5—Cu4—Cl1	121.41 (6)	H17E—C17A—H17F	109.5
Cl6—Cu4—Cl1	112.31 (6)	C17A—O7—H7C	109.5
			
O1—Cu1—Cl1—Cu4	5.31 (12)	Cl2—Cu3—N3—C11	66.2 (3)
N1—Cu1—Cl1—Cu4	−174.19 (15)	Cl6—Cu3—N3—C11	−169.4 (3)
Cl3—Cu1—Cl1—Cu4	−75.77 (7)	Cl4—Cu3—N3—C11	−50.3 (4)
Cl2—Cu1—Cl1—Cu4	87.93 (6)	Cu1—Cu3—N3—C11	71.7 (4)
Cu3—Cu1—Cl1—Cu4	38.51 (4)	Cu2—Cu3—N3—C11	−49.9 (5)
N1—Cu1—O1—Cu2	22 (12)	O1—Cu3—N3—C9	−129 (7)
Cl3—Cu1—O1—Cu2	1.49 (15)	Cl2—Cu3—N3—C9	−165.9 (4)
Cl1—Cu1—O1—Cu2	−124.93 (16)	Cl6—Cu3—N3—C9	−41.5 (4)
Cl2—Cu1—O1—Cu2	118.32 (16)	Cl4—Cu3—N3—C9	77.6 (4)
Cu3—Cu1—O1—Cu2	122.8 (2)	Cu1—Cu3—N3—C9	−160.4 (3)
Cu4—Cu1—O1—Cu2	−117.9 (3)	Cu2—Cu3—N3—C9	77.9 (4)
N1—Cu1—O1—Cu4	140 (12)	C1—N1—C3—C4	52.6 (6)
Cl3—Cu1—O1—Cu4	119.39 (17)	Cu1—N1—C3—C4	−179.1 (4)
Cl1—Cu1—O1—Cu4	−7.03 (15)	Cu1—O1—Cu4—N4	−51 (14)
Cl2—Cu1—O1—Cu4	−123.79 (16)	Cu2—O1—Cu4—N4	68 (14)
Cu3—Cu1—O1—Cu4	−119.3 (3)	Cu3—O1—Cu4—N4	−170 (64)
N1—Cu1—O1—Cu3	−101 (12)	Cu1—O1—Cu4—Cl5	−114.99 (16)
Cl3—Cu1—O1—Cu3	−121.28 (15)	Cu2—O1—Cu4—Cl5	4.25 (14)
Cl1—Cu1—O1—Cu3	112.30 (15)	Cu3—O1—Cu4—Cl5	126.36 (16)
Cl2—Cu1—O1—Cu3	−4.45 (14)	Cu1—O1—Cu4—Cl6	119.92 (17)
Cu4—Cu1—O1—Cu3	119.3 (3)	Cu2—O1—Cu4—Cl6	−120.84 (15)
O1—Cu1—N1—C1	−79 (12)	Cu3—O1—Cu4—Cl6	1.28 (14)
Cl3—Cu1—N1—C1	−59.1 (4)	Cu1—O1—Cu4—Cl1	7.01 (15)
Cl1—Cu1—N1—C1	67.3 (3)	Cu2—O1—Cu4—Cl1	126.25 (15)
Cl2—Cu1—N1—C1	−175.8 (3)	Cu3—O1—Cu4—Cl1	−111.64 (16)
Cu3—Cu1—N1—C1	−179.1 (2)	Cu1—O1—Cu4—Cu2	−119.2 (3)
Cu4—Cu1—N1—C1	59.5 (5)	Cu3—O1—Cu4—Cu2	122.1 (2)
O1—Cu1—N1—C3	154 (12)	Cu2—O1—Cu4—Cu1	119.2 (3)
Cl3—Cu1—N1—C3	174.7 (4)	Cu3—O1—Cu4—Cu1	−118.6 (3)
Cl1—Cu1—N1—C3	−58.9 (4)	Cu1—Cl1—Cu4—O1	−5.31 (11)
Cl2—Cu1—N1—C3	58.0 (4)	Cu1—Cl1—Cu4—N4	174.05 (16)
Cu3—Cu1—N1—C3	54.7 (5)	Cu1—Cl1—Cu4—Cl5	77.07 (7)
Cu4—Cu1—N1—C3	−66.7 (5)	Cu1—Cl1—Cu4—Cl6	−87.93 (5)
C3—N1—C1—C2	−51.8 (6)	Cu1—Cl1—Cu4—Cu2	24.37 (5)
Cu1—N1—C1—C2	179.4 (3)	N2—Cu2—Cu4—O1	−178.9 (3)
Cu1—O1—Cu2—N2	−179 (100)	Cl5—Cu2—Cu4—O1	174.46 (18)
Cu4—O1—Cu2—N2	63 (14)	Cl3—Cu2—Cu4—O1	62.09 (18)
Cu3—O1—Cu2—N2	−58 (14)	Cl4—Cu2—Cu4—O1	−61.05 (17)
Cu1—O1—Cu2—Cl5	114.27 (16)	Cu3—Cu2—Cu4—O1	−33.81 (17)
Cu4—O1—Cu2—Cl5	−4.22 (14)	O1—Cu2—Cu4—N4	−178.8 (3)
Cu3—O1—Cu2—Cl5	−124.95 (16)	N2—Cu2—Cu4—N4	2.3 (3)
Cu1—O1—Cu2—Cl3	−1.48 (14)	Cl5—Cu2—Cu4—N4	−4.3 (3)
Cu4—O1—Cu2—Cl3	−119.97 (15)	Cl3—Cu2—Cu4—N4	−116.7 (3)
Cu3—O1—Cu2—Cl3	119.30 (16)	Cl4—Cu2—Cu4—N4	120.1 (3)
Cu1—O1—Cu2—Cl4	−120.25 (16)	Cu3—Cu2—Cu4—N4	147.4 (3)
Cu4—O1—Cu2—Cl4	121.25 (15)	O1—Cu2—Cu4—Cl5	−174.46 (18)
Cu3—O1—Cu2—Cl4	0.53 (14)	N2—Cu2—Cu4—Cl5	6.7 (2)
Cu1—O1—Cu2—Cu4	118.5 (2)	Cl3—Cu2—Cu4—Cl5	−112.37 (7)
Cu3—O1—Cu2—Cu4	−120.7 (2)	Cl4—Cu2—Cu4—Cl5	124.48 (7)
Cu1—O1—Cu2—Cu3	−120.8 (3)	Cu3—Cu2—Cu4—Cl5	151.73 (6)
Cu4—O1—Cu2—Cu3	120.7 (2)	O1—Cu2—Cu4—Cl6	61.48 (17)
O1—Cu1—Cl2—Cu3	3.41 (11)	N2—Cu2—Cu4—Cl6	−117.4 (2)
N1—Cu1—Cl2—Cu3	−177.50 (13)	Cl5—Cu2—Cu4—Cl6	−124.05 (7)
Cl3—Cu1—Cl2—Cu3	85.82 (6)	Cl3—Cu2—Cu4—Cl6	123.58 (6)
Cl1—Cu1—Cl2—Cu3	−79.46 (6)	Cl4—Cu2—Cu4—Cl6	0.43 (6)
Cu4—Cu1—Cl2—Cu3	−26.63 (5)	Cu3—Cu2—Cu4—Cl6	27.68 (4)
O1—Cu2—N2—C5	−128 (14)	O1—Cu2—Cu4—Cl1	−56.95 (18)
Cl5—Cu2—N2—C5	−61.2 (4)	N2—Cu2—Cu4—Cl1	124.2 (2)
Cl3—Cu2—N2—C5	54.6 (4)	Cl5—Cu2—Cu4—Cl1	117.52 (7)
Cl4—Cu2—N2—C5	173.5 (4)	Cl3—Cu2—Cu4—Cl1	5.14 (6)
Cu4—Cu2—N2—C5	−66.2 (5)	Cl4—Cu2—Cu4—Cl1	−118.00 (6)
Cu3—Cu2—N2—C5	174.9 (3)	Cu3—Cu2—Cu4—Cl1	−90.75 (5)
O1—Cu2—N2—C7	−3 (14)	O1—Cu2—Cu4—Cu1	−35.63 (17)
Cl5—Cu2—N2—C7	64.0 (4)	N2—Cu2—Cu4—Cu1	145.5 (2)
Cl3—Cu2—N2—C7	179.8 (4)	Cl5—Cu2—Cu4—Cu1	138.84 (6)
Cl4—Cu2—N2—C7	−61.4 (4)	Cl3—Cu2—Cu4—Cu1	26.47 (5)
Cu4—Cu2—N2—C7	58.9 (5)	Cl4—Cu2—Cu4—Cu1	−96.68 (5)
Cu3—Cu2—N2—C7	−60.0 (5)	Cu3—Cu2—Cu4—Cu1	−69.43 (3)
C4—O2—C2—C1	−57.5 (6)	N1—Cu1—Cu4—O1	−179.0 (3)
N1—C1—C2—O2	54.6 (6)	Cl3—Cu1—Cu4—O1	−62.9 (2)
Cu1—O1—Cu3—N3	−33 (7)	Cl1—Cu1—Cu4—O1	170.9 (2)
Cu2—O1—Cu3—N3	−154 (7)	Cl2—Cu1—Cu4—O1	58.97 (19)
Cu4—O1—Cu3—N3	86 (7)	Cu3—Cu1—Cu4—O1	35.84 (19)
Cu1—O1—Cu3—Cl2	4.51 (14)	O1—Cu1—Cu4—N4	179.0 (3)
Cu2—O1—Cu3—Cl2	−117.07 (16)	N1—Cu1—Cu4—N4	0.0 (4)
Cu4—O1—Cu3—Cl2	123.29 (16)	Cl3—Cu1—Cu4—N4	116.1 (3)
Cu1—O1—Cu3—Cl6	−120.05 (15)	Cl1—Cu1—Cu4—N4	−10.1 (3)
Cu2—O1—Cu3—Cl6	118.37 (16)	Cl2—Cu1—Cu4—N4	−122.0 (3)
Cu4—O1—Cu3—Cl6	−1.27 (14)	Cu3—Cu1—Cu4—N4	−145.2 (3)
Cu1—O1—Cu3—Cl4	121.06 (15)	O1—Cu1—Cu4—Cl5	66.97 (19)
Cu2—O1—Cu3—Cl4	−0.52 (14)	N1—Cu1—Cu4—Cl5	−112.0 (3)
Cu4—O1—Cu3—Cl4	−120.16 (16)	Cl3—Cu1—Cu4—Cl5	4.12 (6)
Cu2—O1—Cu3—Cu1	−121.6 (2)	Cl1—Cu1—Cu4—Cl5	−122.14 (7)
Cu4—O1—Cu3—Cu1	118.8 (2)	Cl2—Cu1—Cu4—Cl5	125.94 (6)
Cu1—O1—Cu3—Cu2	121.6 (2)	Cu3—Cu1—Cu4—Cl5	102.81 (5)
Cu4—O1—Cu3—Cu2	−119.6 (3)	O1—Cu1—Cu4—Cl6	−62.19 (19)
Cu1—Cl2—Cu3—O1	−3.40 (11)	N1—Cu1—Cu4—Cl6	118.8 (3)
Cu1—Cl2—Cu3—N3	175.76 (13)	Cl3—Cu1—Cu4—Cl6	−125.04 (6)
Cu1—Cl2—Cu3—Cl6	77.27 (7)	Cl1—Cu1—Cu4—Cl6	108.70 (6)
Cu1—Cl2—Cu3—Cl4	−84.38 (6)	Cl2—Cu1—Cu4—Cl6	−3.22 (6)
Cu1—Cl2—Cu3—Cu2	−33.93 (5)	Cu3—Cu1—Cu4—Cl6	−26.35 (4)
N1—Cu1—Cu3—O1	178.5 (3)	O1—Cu1—Cu4—Cl1	−170.9 (2)
Cl3—Cu1—Cu3—O1	61.22 (18)	N1—Cu1—Cu4—Cl1	10.1 (3)
Cl1—Cu1—Cu3—O1	−69.18 (18)	Cl3—Cu1—Cu4—Cl1	126.26 (7)
Cl2—Cu1—Cu3—O1	174.18 (18)	Cl2—Cu1—Cu4—Cl1	−111.92 (7)
Cu4—Cu1—Cu3—O1	−35.70 (17)	Cu3—Cu1—Cu4—Cl1	−135.05 (5)
O1—Cu1—Cu3—N3	178.8 (3)	O1—Cu1—Cu4—Cu2	36.12 (19)
N1—Cu1—Cu3—N3	−2.8 (3)	N1—Cu1—Cu4—Cu2	−142.8 (2)
Cl3—Cu1—Cu3—N3	−120.0 (2)	Cl3—Cu1—Cu4—Cu2	−26.74 (4)
Cl1—Cu1—Cu3—N3	109.6 (2)	Cl1—Cu1—Cu4—Cu2	−153.00 (5)
Cl2—Cu1—Cu3—N3	−7.1 (2)	Cl2—Cu1—Cu4—Cu2	95.09 (5)
Cu4—Cu1—Cu3—N3	143.1 (2)	Cu3—Cu1—Cu4—Cu2	71.95 (3)
O1—Cu1—Cu3—Cl2	−174.18 (18)	O1—Cu2—Cl4—Cu3	−0.38 (10)
N1—Cu1—Cu3—Cl2	4.3 (2)	N2—Cu2—Cl4—Cu3	178.98 (14)
Cl3—Cu1—Cu3—Cl2	−112.96 (7)	Cl5—Cu2—Cl4—Cu3	80.54 (7)
Cl1—Cu1—Cu3—Cl2	116.64 (7)	Cl3—Cu2—Cl4—Cu3	−81.52 (6)
Cu4—Cu1—Cu3—Cl2	150.13 (6)	Cu4—Cu2—Cl4—Cu3	30.79 (4)
O1—Cu1—Cu3—Cl6	61.82 (18)	O1—Cu3—Cl4—Cu2	0.38 (10)
N1—Cu1—Cu3—Cl6	−119.7 (2)	N3—Cu3—Cl4—Cu2	179.77 (14)
Cl3—Cu1—Cu3—Cl6	123.05 (6)	Cl2—Cu3—Cl4—Cu2	82.95 (7)
Cl1—Cu1—Cu3—Cl6	−7.36 (6)	Cl6—Cu3—Cl4—Cu2	−79.84 (6)
Cl2—Cu1—Cu3—Cl6	−124.00 (7)	Cu1—Cu3—Cl4—Cu2	31.09 (4)
Cu4—Cu1—Cu3—Cl6	26.13 (4)	O1—Cu4—N4—C13	−3 (14)
O1—Cu1—Cu3—Cl4	−60.51 (18)	Cl5—Cu4—N4—C13	61.4 (4)
N1—Cu1—Cu3—Cl4	118.0 (2)	Cl6—Cu4—N4—C13	−173.5 (4)
Cl3—Cu1—Cu3—Cl4	0.72 (6)	Cl1—Cu4—N4—C13	−60.5 (4)
Cl1—Cu1—Cu3—Cl4	−129.69 (5)	Cu2—Cu4—N4—C13	64.7 (5)
Cl2—Cu1—Cu3—Cl4	113.67 (7)	Cu1—Cu4—N4—C13	−52.8 (5)
Cu4—Cu1—Cu3—Cl4	−96.20 (5)	O1—Cu4—N4—C15	−128 (14)
O1—Cu1—Cu3—Cu2	−33.44 (17)	Cl5—Cu4—N4—C15	−63.8 (4)
N1—Cu1—Cu3—Cu2	145.0 (2)	Cl6—Cu4—N4—C15	61.3 (4)
Cl3—Cu1—Cu3—Cu2	27.78 (5)	Cl1—Cu4—N4—C15	174.3 (4)
Cl1—Cu1—Cu3—Cu2	−102.63 (4)	Cu2—Cu4—N4—C15	−60.5 (5)
Cl2—Cu1—Cu3—Cu2	140.73 (6)	Cu1—Cu4—N4—C15	−178.0 (3)
Cu4—Cu1—Cu3—Cu2	−69.14 (2)	C2—O2—C4—C3	59.6 (6)
N2—Cu2—Cu3—O1	178.9 (3)	N1—C3—C4—O2	−57.8 (6)
Cl5—Cu2—Cu3—O1	57.70 (18)	O1—Cu4—Cl5—Cu2	−3.26 (11)
Cl3—Cu2—Cu3—O1	−62.30 (18)	N4—Cu4—Cl5—Cu2	177.42 (15)
Cl4—Cu2—Cu3—O1	−179.32 (18)	Cl6—Cu4—Cl5—Cu2	77.75 (7)
Cu4—Cu2—Cu3—O1	35.81 (18)	Cl1—Cu4—Cl5—Cu2	−85.38 (7)
O1—Cu2—Cu3—N3	178.9 (3)	Cu1—Cu4—Cl5—Cu2	−35.74 (5)
N2—Cu2—Cu3—N3	−2.2 (3)	O1—Cu2—Cl5—Cu4	3.27 (11)
Cl5—Cu2—Cu3—N3	−123.4 (2)	N2—Cu2—Cl5—Cu4	−176.04 (14)
Cl3—Cu2—Cu3—N3	116.6 (2)	Cl3—Cu2—Cl5—Cu4	85.24 (6)
Cl4—Cu2—Cu3—N3	−0.4 (2)	Cl4—Cu2—Cl5—Cu4	−77.28 (7)
Cu4—Cu2—Cu3—N3	−145.3 (2)	Cu3—Cu2—Cl5—Cu4	−25.09 (5)
O1—Cu2—Cu3—Cl2	65.01 (18)	C7—N2—C5—C6	53.4 (7)
N2—Cu2—Cu3—Cl2	−116.1 (2)	Cu2—N2—C5—C6	−178.7 (4)
Cl5—Cu2—Cu3—Cl2	122.72 (6)	O1—Cu4—Cl6—Cu3	−0.96 (11)
Cl3—Cu2—Cu3—Cl2	2.71 (6)	N4—Cu4—Cl6—Cu3	178.91 (15)
Cl4—Cu2—Cu3—Cl2	−114.30 (7)	Cl5—Cu4—Cl6—Cu3	−82.48 (7)
Cu4—Cu2—Cu3—Cl2	100.82 (5)	Cl1—Cu4—Cl6—Cu3	82.00 (6)
O1—Cu2—Cu3—Cl6	−62.99 (18)	Cu2—Cu4—Cl6—Cu3	−32.26 (5)
N2—Cu2—Cu3—Cl6	115.9 (2)	Cu1—Cu4—Cl6—Cu3	30.15 (4)
Cl5—Cu2—Cu3—Cl6	−5.29 (6)	O1—Cu3—Cl6—Cu4	0.96 (11)
Cl3—Cu2—Cu3—Cl6	−125.29 (6)	N3—Cu3—Cl6—Cu4	−177.65 (13)
Cl4—Cu2—Cu3—Cl6	117.69 (6)	Cl2—Cu3—Cl6—Cu4	−80.61 (7)
Cu4—Cu2—Cu3—Cl6	−27.19 (4)	Cl4—Cu3—Cl6—Cu4	80.69 (6)
O1—Cu2—Cu3—Cl4	179.32 (18)	Cu1—Cu3—Cl6—Cu4	−30.13 (4)
N2—Cu2—Cu3—Cl4	−1.8 (2)	Cu2—Cu3—Cl6—Cu4	31.00 (4)
Cl5—Cu2—Cu3—Cl4	−122.98 (7)	C8—O3—C6—C5	59.0 (7)
Cl3—Cu2—Cu3—Cl4	117.02 (7)	N2—C5—C6—O3	−57.1 (8)
Cu4—Cu2—Cu3—Cl4	−144.88 (5)	C5—N2—C7—C8	−54.6 (6)
O1—Cu2—Cu3—Cu1	35.07 (18)	Cu2—N2—C7—C8	176.3 (4)
N2—Cu2—Cu3—Cu1	−146.0 (2)	C6—O3—C8—C7	−60.3 (6)
Cl5—Cu2—Cu3—Cu1	92.78 (5)	N2—C7—C8—O3	59.2 (6)
Cl3—Cu2—Cu3—Cu1	−27.23 (4)	C11—N3—C9—C10	−50.0 (6)
Cl4—Cu2—Cu3—Cu1	−144.24 (5)	Cu3—N3—C9—C10	−179.9 (4)
Cu4—Cu2—Cu3—Cu1	70.88 (3)	C12—O4—C10—C9	−61.3 (6)
O1—Cu1—Cl3—Cu2	−1.11 (11)	N3—C9—C10—O4	56.8 (6)
N1—Cu1—Cl3—Cu2	179.20 (14)	C9—N3—C11—C12	50.6 (6)
Cl1—Cu1—Cl3—Cu2	80.17 (7)	Cu3—N3—C11—C12	−178.5 (4)
Cl2—Cu1—Cl3—Cu2	−83.52 (6)	C10—O4—C12—C11	61.0 (6)
Cu3—Cu1—Cl3—Cu2	−32.03 (5)	N3—C11—C12—O4	−57.2 (6)
Cu4—Cu1—Cl3—Cu2	30.21 (5)	C15—N4—C13—C14	−51.3 (6)
O1—Cu2—Cl3—Cu1	1.11 (11)	Cu4—N4—C13—C14	−178.0 (4)
N2—Cu2—Cl3—Cu1	−178.92 (15)	C16—O5—C14—C13	−61.9 (7)
Cl5—Cu2—Cl3—Cu1	−81.42 (6)	N4—C13—C14—O5	57.4 (7)
Cl4—Cu2—Cl3—Cu1	82.34 (6)	C13—N4—C15—C16	52.0 (6)
Cu4—Cu2—Cl3—Cu1	−30.41 (5)	Cu4—N4—C15—C16	178.8 (4)
Cu3—Cu2—Cl3—Cu1	30.99 (5)	C14—O5—C16—C15	62.0 (7)
O1—Cu3—N3—C11	103 (7)	N4—C15—C16—O5	−57.9 (7)

### Hydrogen-bond geometry (Å, º)

**Table:**

*D*—H···*A*	*D*—H	H···*A*	*D*···*A*	*D*—H···*A*
N1—H1···O5^i^	0.84 (3)	2.22 (3)	3.060 (6)	177 (5)
N2—H2···O6^ii^	0.84 (3)	2.18 (3)	2.987 (8)	161 (5)
N3—H3···O8^iii^	0.84 (3)	2.05 (3)	2.871 (6)	167 (5)
N4—H4···O7^iv^	0.84 (3)	2.30 (3)	3.121 (12)	165 (5)
O7—H7*C*···O4	0.82	1.92	2.531 (12)	130
O8—H8···O2	0.82	1.91	2.724 (6)	173
O6—H6*C*···Cl4^v^	0.82	2.39	3.209 (7)	177

Symmetry codes: (i) *x*+1/2, −*y*+1/2, *z*−1/2; (ii) *x*−1/2, −*y*+1/2, *z*−1/2; (iii) −*x*, −*y*, −*z*; (iv) *x*−1, *y*, *z*; (v) −*x*+3/2, *y*−1/2, −*z*+1/2.
